# Estimated Dietary Lead Exposure Through Aquatic Animal Consumption and Health Symptoms in a Lake-Dependent Community: Evidence from Nong Han Lake, Northeast Thailand

**DOI:** 10.3390/ijerph23070899

**Published:** 2026-07-12

**Authors:** Birabongse Hardthakwong, Kulthida Y. Kopolrat, Panchamapohn Rattanahon, Sribud Srichaijaroonpong, Piyapong Chaiyasarn, Narita Fakkaew, Nutta Taneepanichskul, Ratanee Kammoolkon

**Affiliations:** 1 Faculty of Public Health, Kasetsart University, Chalermphrakiat Sakon Nakhon Province Campus, Sakon Nakhon 47000, Thailand; birabongse.h@ku.th (B.H.); kulthida.y@ku.th (K.Y.K.); panchamapohn.r@ku.th (P.R.); sribud.s@ku.th (S.S.); piyapong.chai@ku.th (P.C.); narita.fak@ku.th (N.F.); 2 College of Public Health Sciences, Chulalongkorn University, Bangkok 10330, Thailand; nutta.t@chula.ac.th

**Keywords:** lead contamination, aquatic animals, estimated dietary Pb intake (EDI), self-reported health symptoms, health risk assessment, lake-dependent community

## Abstract

**Highlights:**

**Public health relevance—How does this work relate to a public health issue?**

- Species-specific lead (Pb) concentrations in freshwater fish, shrimp, and snails were integrated with dietary consumption data to estimate individual dietary Pb exposure.
- The study examined dietary Pb exposure and self-reported health symptoms in a lake-dependent community with frequent freshwater aquatic food consumption.

**Public health significance—Why is this work of significance to public health?**

- Median estimated dietary Pb intake was 0.1004 µg/kg body weight/day, although Pb concentrations in all aquatic species remained below the FAO/WHO Codex maximum level.
- Large portion sizes and long-term aquatic animal consumption were independently associated with self-reported health symptoms.

**Public health implications—What are the key implications or messages for practitioners, policy makers and/or researchers in public health?**

- Dietary exposure assessment should complement environmental monitoring when evaluating potential Pb exposure in freshwater-dependent communities.
- Tailored health education, dietary risk communication, and biomonitoring may strengthen exposure prevention and support public health decision-making.

**Abstract:**

Lead (Pb) is a persistent environmental contaminant and a major public health concern. This cross-sectional study investigated self-reported health symptoms and estimated dietary Pb exposure from aquatic animal consumption among residents living around Nong Han Lake, Thailand. Sociodemographic characteristics, dietary behaviors, and health symptoms were collected using a structured questionnaire. Pb concentrations were measured in three commonly consumed aquatic species, and species-specific estimated dietary Pb intake (EDI) was calculated from Pb concentrations and reported consumption patterns. Multivariable logistic regression identified factors associated with symptom reporting. Pb concentrations in all aquatic species were below the permissible limit (0.3 mg/kg wet weight). Median EDI was 0.1004 µg/kg bw/day (IQR: 0.0645–0.1785). Musculoskeletal pain (79.73%) and joint and muscle pain (71.62%) were the most commonly reported symptoms. This study found that female sex (aOR = 2.62; 95% CI: 1.099–6.249; *p* = 0.030), a lack of lead hazard training (aOR = 3.39; 95% CI: 1.373–8.418; *p* = 0.008), consumption of more than 300 g of aquatic animals per meal (aOR = 2.47; 95% CI: 1.230–4.960; *p* = 0.011), and long-term consumption exceeding 30 years (aOR = 2.16; 95% CI: 1.065–4.398; *p* = 0.033) were each independently associated with symptom reporting. Estimated dietary Pb intake (EDI) was not significantly associated with symptoms after multivariable adjustment (aOR = 1.30; 95% CI: 0.657–2.578; *p* = 0.450). These findings suggest that consuming large portions of aquatic animals, consuming aquatic animals over prolonged periods, and having limited awareness of lead-related hazards are potential pathways for dietary Pb exposure in this lake-dependent community.

## 1. Introduction

Aquatic animals are important sources of essential nutrients but may also accumulate environmental contaminants, raising concerns about potential health impacts on consumers [1]. Lead (Pb) is a persistent toxic heavy metal of major global public health concern. Dietary intake is a primary route of chronic exposure, with fish and other aquatic animals serving as a key exposure pathway in communities reliant on freshwater resources [2,3]. Pb accumulates in aquatic environments through industrial, agricultural, and other anthropogenic activities and can bioaccumulate in aquatic organisms, particularly sediment-associated species. Although Pb generally exhibits limited biomagnification compared with persistent organic contaminants, its accumulation in edible aquatic organisms is primarily influenced by environmental contamination, species-specific feeding behavior, and habitat characteristics, thereby representing an important dietary exposure pathway for humans [4,5,6]. Chronic dietary exposure to Pb has been associated with adverse effects on the nervous, gastrointestinal, hematological, renal, and cardiovascular systems, with commonly reported symptoms including headache, fatigue, musculoskeletal pain, and impaired concentration [7,8]. Prolonged exposure has been linked to hypertension, cognitive impairment, and reproductive toxicity [9,10]. Chronic low-level lead exposure has been estimated to account for approximately 5.5 million cardiovascular deaths and a loss of 765 million IQ points in children annually, with the greatest burden falling on low- and middle-income countries [11]. The World Health Organization (WHO) identifies Pb as a chemical of major public health concern because of its associations with neurological impairment, cardiovascular disease, chronic kidney disease, and premature mortality. Recent risk assessments indicate that no level of Pb exposure can be considered completely safe. Consequently, WHO and other international expert bodies recommend the use of benchmark dose lower confidence limits (BMDLs), derived from epidemiological evidence, to characterize health risks associated with low-level Pb exposure, particularly neurodevelopmental effects in children and cardiovascular outcomes in adults. These findings indicate that adverse health effects may occur even at relatively low exposure levels, emphasizing the importance of minimizing dietary Pb exposure whenever possible [12]. Economic modelling analyses have estimated the combined global cost of lead-attributable cardiovascular mortality and cognitive loss at approximately $6 trillion in 2019, underscoring the substantial societal burden of even low-level exposure [13].

In Thailand, community-based studies near contaminated water bodies have reported symptoms consistent with possible chronic low-level Pb exposure, suggesting subclinical health effects may be widespread but underrecognized [14]. Low-level lead exposure has been independently associated with increased long-term cardiovascular and all-cause mortality [11,15], underscoring its role as a silent contributor to chronic disease burden. Lead-related health impacts also carry substantial economic consequences, including direct medical costs, productivity loss, and long-term disability, particularly in resource-limited settings [16,17,18].

In Thailand, freshwater ecosystems are vital for food security and local livelihoods, particularly in northeastern communities [19]. Nong Han Lake, Sakon Nakhon Province, is one of the largest wetlands in the region and a primary source of aquatic animals for surrounding communities [20]. A systematic review of heavy metal contamination in Thai freshwater environments reported the presence of Pb and other metals in aquatic organisms and sediments, raising concerns about potential dietary exposure and food safety [21]. However, epidemiological evidence examining associations between aquatic animal consumption and self-reported health symptoms in community settings remains limited.

Given the continued reliance on locally sourced aquatic animals and the absence of routine blood lead monitoring in this population, assessing potential health impacts associated with estimated dietary Pb exposure is an important public health priority. Therefore, this study aimed to determine the prevalence of self-reported health symptoms among residents living around Nong Han Lake and to identify factors associated with symptom reporting in relation to aquatic animal consumption patterns and estimated dietary Pb intake.

## 2. Materials and Methods

### 2.1. Study Design and Participants

**Figure 1:**
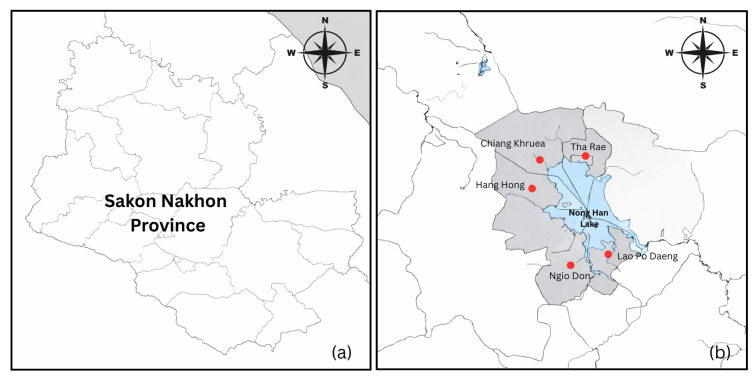
Study area (**a**) and sampling locations (**b**) around Nong Han Lake, Sakon Nakhon Province, Thailand. Administrative boundaries were adapted from GADM (version 4.1), and the figure was prepared and annotated by the authors [23].

This cross-sectional analytic study was conducted between June and August 2025 in five subdistricts (Tha Rae, Lao Po Daeng, Ngio Don, Hang Hong, and Chiang Khruea) of Sakon Nakhon Province, Thailand, as shown in Figure 1. The sample size was calculated to estimate the proportion of the population by the following Equation (1) [22].

(1) $$n=\frac{Z_{\alpha /2}^{2}\;[p(1\;-\;p)]}{e^{2}}$$

The sample size was calculated using the standard formula for estimating a population proportion. For a 95% confidence interval, the Z value was set at 1.96, and the allowable margin of error (e) was set at 0.05, as determined by the researchers. The expected prevalence (p) was set at 9%, based on findings from previous studies [24]. Based on these parameters, the required sample size was estimated to be 139 participants. To account for a potential data loss or incomplete responses of 15%, the final target sample size was increased to 148 participants.

The study employed a purposive sampling strategy to recruit 148 residents living around Nong Han Lake who met predefined eligibility criteria. Participants were adults aged 18 years or older who had resided in the area for at least one year, reported consuming aquatic animals from the lake on a regular basis, and were able to provide informed consent and complete the interview. Individuals with severe illness or cognitive impairment that could affect questionnaire participation were excluded. To enhance representativeness and reduce selection bias, recruitment was conducted across five sub-districts surrounding the lake, covering diverse demographic and occupational backgrounds. When more than one eligible individual resided in the same household, one participant was selected using a simple random approach to minimize intra-household selection bias. Clear inclusion criteria, standardized procedures, and geographically distributed recruitment were implemented to enhance internal validity and reduce potential sampling bias. Ethical approval for this study was obtained from Kasetsart University’s accredited Human Ethics Committee (KUREC-COE68/015 on 23 May 2025). Written informed consent was obtained from all participants, who voluntarily agreed to participate in the research.

### 2.2. Data Collection and Questionnaire Administration

Sociodemographic and health-related data were collected through physical interaction using a structured questionnaire administered and conducted by trained research assistants. The instrument was adapted from the medical history and physical examination tool for individuals at risk of lead poisoning developed by the Occupational and Environmental Diseases Division, Department of Disease Control, Ministry of Public Health, Thailand (2020) [25]. Data were collected using a structured questionnaire comprising three sections. The first section obtained sociodemographic information, including age, sex, education level, occupation, smoking status, alcohol consumption, duration of residence, and history of regular health check-ups, to characterize participants and control for potential confounders. The second section assessed potential lead exposure-related factors, including portion size of consumed aquatic animals, frequency, and duration of aquatic animal consumption from Nong Han Lake. Additional items covered occupational exposure, history of blood lead level (BLL) testing, and participation in educational training on lead hazards, capturing both environmental and behavioral exposure pathways. The third section evaluated self-reported health symptoms potentially associated with chronic lead exposure within the past six months, such as musculoskeletal pain, headache, loss of appetite, nausea and vomiting, fatigue, numbness, abdominal discomfort. Responses were recorded as binary variables (yes/no) for statistical analysis. The questionnaire was developed based on a review of the relevant literature and demonstrated good internal consistency (Cronbach’s alpha = 0.89). Content validity was assessed by three subject-matter experts.

To minimize information bias, all research assistants underwent standardized training and followed a structured questionnaire protocol with predefined wording and neutral prompting techniques. To reduce social desirability bias, interviews were conducted individually in a private setting, participants were assured of confidentiality and anonymity, and they were informed that responses would not affect access to services or benefits. Written informed consent was obtained prior to participation. All complete questionnaires were reviewed for completeness and accuracy before data entry and analysis to ensure data quality and consistency.

### 2.3. Determination of Lead (Pb) in Aquatic Animals

Aquatic animal samples were collected from five monitoring stations in Nong Han Lake during the dry season (June–July 2025). Three commonly consumed species, *Macrobrachium lanchesteri*, *Oreochromis niloticus*, and *Filopaludina martensi* were collected from the same locations and taxonomically confirmed, and only mature edible-sized individuals were included. Edible tissues were dissected, homogenized, and stored at −20 °C prior to analysis. Approximately 1.00 g (fresh weight) of aquatic animal tissue was digested using closed-vessel microwave digestion with HNO_3_ and H_2_O_2_. Digests were diluted to 10 mL with Milli-Q water and analyzed for Pb using an Agilent 5800 inductively coupled plasma optical emission spectrometer (ICP-OES; Agilent Technologies, Santa Clara, CA, USA). Calibration was performed using analytical-grade Pb standards, and quality assurance/quality control included reagent blanks and certified reference material (NRCC-DORM-2). The method detection limit was determined in accordance with AOAC guidelines. Lead concentrations were quantified by ICP-OES. The analytical method showed excellent linearity over the concentration range of 0.005–0.6 mg/L (R^2^ = 0.9999), with a working range of 10–400 μg/L. The limit of detection (LOD) and limit of quantification (LOQ) were 0.0005 mg/L and 0.002 mg/L, respectively. All measured Pb concentrations exceeded the LOD; therefore, no left-censored observations were present, and no substitution or imputation procedures were required. Descriptive statistics, including the mean, standard deviation, and maximum Pb concentrations, were calculated directly from the measured values. Pb concentrations were compared with the permissible limit established by the Food and Agriculture Organization of the United Nations (FAO)/World Health Organization (WHO) (FAO/WHO = 0.3 mg/kg) [26].

### 2.4. Estimated Dietary Pb Intake Assessment

Estimated dietary Pb intake (EDI) from aquatic animal consumption was calculated by integrating measured Pb concentrations in aquatic animal samples with participants’ self-reported consumption patterns, using Equation (2), as recommended by the United States Environmental Protection Agency (USEPA, 2004) [27,28].

(2) $$\mathrm{EDI}=\frac{C\;\times \;\mathrm{IR}}{\mathrm{BW}}$$

where C represents the species-specific mean Pb concentration (mg/kg wet weight) measured in fish, freshwater shrimp, and freshwater snails; IR is the species-specific ingestion rate (kg/day); and BW is the participant’s self-reported body weight (kg).

The ingestion rate (IR) for each aquatic species was estimated from participants’ self-reported portion size and consumption frequency obtained through the questionnaire. Categorical portion sizes were converted into representative consumption amounts of 50, 150, 250, and 300 g/meal for the categories <100, 100–200, 200–300, and >300 g/meal, respectively. Consumption frequency was converted into meals/day using predefined conversion factors of 3.000 (every meal), 1.000 (at least one meal/day), 0.357 (2–3 meals/week), 0.050 (1–2 meals/month), 0.011 (special occasions; approximately four meals/year), and 0 (never consumed). The daily ingestion rate (IR, kg/day) was calculated as

(3) $$\mathrm{IR}=\frac{\left(portion\;size\;[g/meal]\times meals/day\right)}{1000}$$

Species-specific EDI values were then calculated separately for fish, freshwater shrimp, and freshwater snails by combining the corresponding Pb concentration with the respective ingestion rate for each species. The total EDI for each participant was calculated by summing the species-specific EDI values across all aquatic species consumed. To facilitate multivariable logistic regression analysis and ensure adequate sample size within each exposure category, total EDI values were dichotomized into low- and high-exposure groups using the median EDI of the study population as the cut-off value.

### 2.5. Data Analysis

Data were analyzed using IBM SPSS Statistics version 26.0 (IBM Corp., Armonk, NY, USA). Descriptive statistics were used to summarize participant characteristics and the prevalence of self-reported health symptoms. Categorical variables were presented as frequencies (n) and percentages (%), while estimated dietary Pb intake (EDI) was summarized using the median, interquartile range (IQR), and range. The prevalence of each health symptom was estimated as proportions with corresponding 95% confidence intervals (CIs). For regression analyses, the binary outcome variable represented the presence of any self-reported health symptom, defined as reporting at least one symptom included in the study questionnaire (yes/no). Estimated dietary Pb intake was calculated for each participant and subsequently categorized into high and low EDI groups based on the median value. Thus, multivariable models estimated associations with overall symptom reporting rather than individual symptoms.

Associations between personal characteristics, lead exposure-related factors, and health symptoms were examined using logistic regression analysis. Potential confounders were identified a priori based on relevant literature and epidemiological considerations. Variables with a univariate association (*p* < 0.20), together with those considered biologically relevant, were considered for multivariable analysis. Rather than fitting a single fully adjusted model for all predictors, separate multivariable logistic regression models were constructed for each primary exposure variable. Covariates were selected a priori to minimize over-adjustment by avoiding the simultaneous inclusion of conceptually related exposure variables, such as estimated dietary Pb intake and duration of aquatic animal consumption. The covariates included in each adjusted model are specified in the footnotes of Table 3. Multicollinearity among candidate covariates was assessed using the variance inflation factor (VIF). All VIF values ranged from 1.029 to 1.290, indicating no evidence of problematic multicollinearity; therefore, all candidate covariates remained eligible for inclusion in the multivariable analyses. Final adjusted models were specified according to the predefined modelling strategy for each exposure variable. Model fit was evaluated using the Hosmer–Lemeshow goodness-of-fit test. Adjusted odds ratios (aORs) with 95% confidence intervals (CIs) were reported, and a two-sided *p* < 0.05 was considered statistically significant.

## 3. Results

### 3.1. General Characteristics

A total of 148 residents participated in the study. The majority were female (67.6%) and married (71.6%), with most aged over 35 years (81.8%). Approximately one-third were engaged in agricultural occupations (34.5%), and 42.6% had no formal education or primary education. Most participants reported no history of smoking (77.0%) or alcohol consumption (88.5%), and 56.8% underwent regular annual health check-ups. A large proportion had previously received educational training on the hazards and toxic effects of lead (77.7%) and had undergone blood lead level (BLL) testing (20.9%). Regarding dietary behavior, 45.9% consumed more than 300 g of aquatic animals per meal, 28.4% reported consumption of more than one meal per day, and 47.3% had consumed aquatic animals from Nong Han Lake for more than 30 years. Overall, the study population was predominantly middle-aged or older adults with long-term and relatively high-level aquatic animal consumption, indicating a potentially sustained dietary exposure pathway relevant to environmental lead risk in this community (Table 1).

### 3.2. Lead (Pb) Concentrations in Aquatic Animals

**Figure 2:**
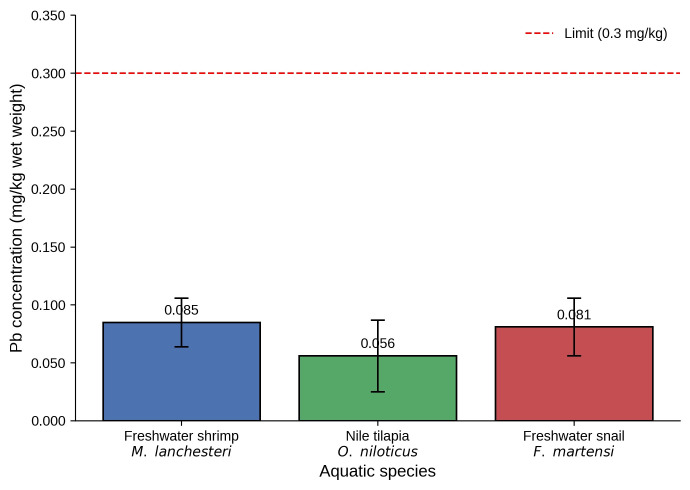
Mean Pb concentrations (mg/kg wet weight) in fish (*Oreochromis niloticus*), freshwater shrimp (*Macrobrachium lanchesteri*), and freshwater snails (*Filopaludina martensi*) collected from five sampling sites around Nong Han Lake. Error bars represent standard deviations. Species-specific mean Pb concentrations were used in the estimation of dietary Pb intake (EDI). Detailed descriptive statistics are provided in Supplementary Table S1.

Figure 2 presents the species-specific mean Pb concentrations in the three aquatic species most commonly consumed by the study population. Freshwater shrimp (*Macrobrachium lanchesteri*) had the highest mean Pb concentration (0.085 mg/kg wet weight), followed by freshwater snails (*Filopaludina martensi*; 0.081 mg/kg) and Nile tilapia (*Oreochromis niloticus*; 0.056 mg/kg). Although Pb accumulation varied among species, all mean concentrations remained below the FAO/WHO Codex maximum level for fish products (0.3 mg/kg wet weight). However, compliance with this regulatory standard should not be interpreted as indicating the absence of potential health risk, particularly among populations with long-term dependence on freshwater aquatic foods.

Species and site-specific descriptive statistics, including mean, standard deviation, minimum, and maximum Pb concentrations, are provided in Supplementary Table S1. These data show modest spatial variation in Pb concentrations across the five sampling sites, indicating heterogeneous Pb distribution within Nong Han Lake. The species-specific mean Pb concentrations were subsequently combined with species-specific ingestion rates derived from participants’ reported dietary consumption patterns to estimate individual estimated dietary Pb intake (EDI). Total EDI was calculated as the sum of the species-specific EDIs from Nile tilapia, freshwater shrimp, and freshwater snails.

### 3.3. Estimated Dietary Pb Intake

Estimated dietary Pb intake (EDI) was calculated using species-specific Pb concentrations and corresponding ingestion rates for fish, freshwater shrimp, and freshwater snails. The total EDI for each participant was obtained by summing the species-specific EDI values. The mean estimated dietary Pb intake was 0.191 ± 0.297 µg/kg bw/day, with a median of 0.1004 µg/kg bw/day (IQR: 0.0645–0.1785). Based on the median value, participants were classified into low and high EDI groups for subsequent analyses.

### 3.4. Types and Prevalence of Health Symptoms

Among the 148 participants, the prevalence of self-reported health symptoms varied across categories (Table 2). The most frequently reported symptoms were musculoskeletal pain (79.73%; 95% CI: 73.1–86.2) and joint and muscle pain (71.62%; 95% CI: 64.2–78.9). Headache was reported by 58.78% (95% CI: 50.7–66.8), and irritability by 54.73% (95% CI: 46.6–62.8). Moderate prevalence levels were observed for restlessness or poor concentration (43.20%; 95% CI: 35.1–51.3), fatigue (39.86%; 95% CI: 31.9–48.2), and constipation (32.43%; 95% CI: 24.8–40.0). Gastrointestinal symptoms—including loss of appetite (22.97%; 95% CI: 16.1–29.8), nausea and vomiting (16.89%; 95% CI: 10.7–22.9), and severe intermittent abdominal pain (14.86%; 95% CI: 9.0–20.6)—were less common. Pallor (8.78%; 95% CI: 4.1–13.3) and lethargy (7.43%; 95% CI: 3.1–11.7) were infrequently reported, while seizure was rare (0.68%). Overall, the predominance of musculoskeletal and nonspecific neurological symptoms suggests a pattern consistent with chronic low-level exposure rather than acute lead toxicity, warranting further investigation of cumulative environmental and dietary exposure pathways in this population.

### 3.5. Factors Associated with Any Self-Reported Health Symptoms

Multivariable logistic regression analysis identified several factors associated with self-reported health symptoms among residents living around Nong Han Lake are presented in Table 3. The model demonstrated adequate fit (Hosmer–Lemeshow χ^2^ = 1.398, df = 5, *p* = 0.925). Female participants were significantly more likely to report symptoms than males (aOR = 2.62, 95% CI: 1.099–6.249, *p*-value = 0.030). Participants who had never received educational training regarding the hazards and toxic effects of lead showed significantly higher odds of symptom reporting compared with those who had received such training (aOR = 3.39, 95% CI: 1.373–8.418, *p*-value = 0.008). Similarly, individuals consuming more than 300 g of aquatic animals per meal had approximately 2.5-fold higher odds of reporting symptoms than those consuming smaller portions (aOR = 2.47, 95% CI: 1.230–4.960, *p*-value = 0.011). Long-term consumption of aquatic animals (>30 years) was also significantly associated with symptom reporting (aOR = 2.16, 95% CI: 1.065–4.398, *p*-value = 0.033). Participants with high estimated dietary Pb intake (EDI) had 1.30 times higher odds of reporting health symptoms than those with low EDI; however, this association was not statistically significant after adjustment for potential confounders (aOR = 1.15, 95% CI: 0.568–2.236, *p*-value = 0.698).

## 4. Discussion

This study assessed the potential dietary Pb exposure among residents living around Nong Han Lake by integrating species-specific Pb concentrations in commonly consumed aquatic animals with individual-level consumption patterns. Although the mean Pb concentrations in fish, freshwater shrimp, and freshwater snails were all below the FAO/WHO Codex maximum level for Pb in fish muscle (0.30 mg/kg wet weight), Pb concentrations varied across sampling sites, indicating spatial heterogeneity in environmental contamination [26]. This spatial variation suggests that dietary Pb exposure may differ by location, particularly among individuals who frequently consume locally sourced aquatic animals. Similar spatial variability has been reported in other freshwater ecosystems, where Pb concentrations generally remain within regulatory limits but are influenced by sediment resuspension, agricultural runoff, and other anthropogenic activities [29,30]. These findings indicate that exposure assessment should consider not only overall Pb concentrations but also spatial variation in contamination and long-term dietary consumption patterns, particularly in communities that rely heavily on freshwater aquatic resources.

The high prevalence of musculoskeletal and neurological symptoms observed in this study is consistent with previous reports from populations exposed to low-level environmental contaminants, although the cross-sectional design precludes causal inference. The predominance of musculoskeletal symptoms is consistent with the findings of Sangwijit et al. [31], who reported a high prevalence of musculoskeletal disorders and headache among lead-exposed e-waste workers. In the present study, larger portion sizes (>300 g per meal) and long-term consumption of aquatic animals (>30 years) were independently associated with symptom reporting, suggesting that cumulative dietary exposure may be more relevant than short-term exposure. Experimental studies have shown that chronic Pb exposure induces oxidative stress, disrupts calcium homeostasis, and impairs neuromuscular and neurobehavioral function [24,32,33]. Although these mechanisms support the biological plausibility of our findings, the observed associations should be interpreted as reflecting potential cumulative dietary exposure rather than direct evidence of Pb-specific health effects.

The association between symptom reporting and consumption of more than 300 g of aquatic animals per meal further supports the importance of dietary dose in chronic Pb exposure. Recent studies suggest that the total amount of contaminated food consumed is a stronger determinant of internal Pb burden than consumption frequency alone [34,35]. Han et al. (2021) reported a mean dietary Pb intake of 0.067 µg/kg bw/day among fish consumers in coastal China and demonstrated that individuals with larger portion sizes accumulated greater Pb burdens over time [36]. Similarly, consumption frequency was not significantly associated with symptom reporting in the present study, whereas larger portion sizes and prolonged consumption duration remained significant predictors. These findings emphasize that long-term dietary habits may contribute more to cumulative Pb exposure than the frequency of consumption alone. Chronic Pb exposure has been linked to musculoskeletal and neurological dysfunction through oxidative stress, altered calcium signaling, and impaired neurotransmitter activity [37,38].

Participants with high estimated dietary Pb intake (EDI) reported symptoms slightly more frequently than those with low EDI; however, this association was not statistically significant after multivariable adjustment (aOR = 1.15, 95% CI: 0.568–2.326; *p* = 0.698). The wide confidence interval indicates limited precision, which may reflect the relatively small sample size and exposure misclassification associated with estimating EDI from categorical self-reported dietary data [39]. Residual overlap between EDI and consumption-related variables included in the multivariable model may also have attenuated the observed association. Although the direction of the association was consistent with previous studies conducted in aquatic food-dependent populations [40], these findings should be interpreted cautiously because a type II error cannot be excluded. Future studies incorporating biomarker-based exposure assessment, such as blood lead levels, would improve exposure characterization and reduce measurement uncertainty.

Female participants had significantly greater odds of reporting health symptoms than males. This finding may reflect sex-related differences in Pb toxicokinetics and susceptibility. Previous studies have shown that women may have higher circulating Pb concentrations and distinct immunological responses to chronic Pb exposure than men, potentially increasing vulnerability to adverse health effects at comparable exposure levels [41,42]. Experimental evidence further suggests that Pb disrupts calcium homeostasis and mitochondrial function in skeletal muscle, with hormonal regulation contributing to sex-specific toxicokinetic differences [43]. Behavioral factors may also contribute, as women are generally more likely to recognize and report chronic health symptoms in population-based surveys.

Participants who had never received training on lead hazards were also more likely to report symptoms. This finding may indicate lower awareness of exposure prevention and risk reduction rather than differences in Pb exposure itself. Previous studies have similarly shown that health education improves recognition of environmental hazards and promotes protective behaviors, although it does not necessarily reduce exposure levels [44,45]. Likewise, a recent community-based study among fishing communities around Lake Victoria reported limited public awareness of heavy metal contamination in commonly consumed fish, emphasizing persistent gaps in environmental health literacy [46]. These findings support strengthening community education alongside practical interventions that reduce dietary exposure to contaminated aquatic foods.

Participants without previous blood lead level (BLL) testing were more likely to report symptoms in the univariable analysis, although this association was no longer significant after multivariable adjustment. This finding suggests that the initial association was largely explained by other participant characteristics included in the model. Nevertheless, limited access to biomonitoring may reduce opportunities for early identification of Pb exposure and timely preventive interventions. Community-based biomonitoring programs have been shown to improve exposure awareness and facilitate early detection of subclinical Pb exposure [47].

An additional limitation is the lack of information on co-exposure to other potentially toxic metals, particularly arsenic (As), cadmium (Cd), and mercury (Hg), which may occur in freshwater agricultural ecosystems through agricultural runoff and sediment accumulation [48]. Several symptoms reported in this study, including fatigue, musculoskeletal pain, and headache, are non-specific and overlap with the health effects of these contaminants. Cadmium has been associated with musculoskeletal disorders and renal tubular dysfunction [49], whereas chronic arsenic exposure has been linked to peripheral neuropathy and fatigue [50]. Because residents of lake-dependent communities may be exposed to multiple contaminants through the same dietary pathway, additive or synergistic effects cannot be excluded. Consequently, the observed associations should be interpreted as reflecting potential cumulative environmental exposure rather than Pb exposure alone. Future studies should simultaneously quantify multiple contaminants and apply mixture-based analytical approaches, such as Weighted Quantile Sum (WQS) regression or Bayesian Kernel Machine Regression (BKMR), to better characterize combined health effects [51].

Although Pb concentrations in aquatic animals remained below the FAO/WHO Codex reference value, this regulatory standard is intended for food safety management rather than direct health risk characterization. Therefore, concentrations below the Codex maximum level do not necessarily preclude potential health concerns, particularly in populations with long-term, high-level consumption of freshwater aquatic foods. Experimental studies have demonstrated that environmentally relevant Pb exposure induces oxidative stress, disrupts calcium signaling, and impairs neurobehavioral function even at relatively low concentrations [52,53]. These mechanisms provide biological plausibility for the observed associations but do not establish causality. Because the reported symptoms are non-specific and may be influenced by multiple environmental and occupational exposures, the findings should be interpreted with appropriate caution. Integrating environmental monitoring with biomarker surveillance would strengthen exposure assessment and improve public health decision-making in lake-dependent communities [54].

Overall, the present findings indicate that consuming large portions of aquatic animals and maintaining this dietary pattern over many years may increase the potential for cumulative dietary Pb exposure, despite Pb concentrations remaining below current regulatory limits. These associations should be considered exploratory rather than evidence of a direct Pb-specific health effect, particularly given the possibility of co-exposure to other environmental contaminants and uncertainties in Pb bioavailability among aquatic species and food preparation methods. The species-specific EDI approach adopted in this study provides a more refined estimate of dietary exposure than environmental Pb concentrations alone; however, its association with symptom reporting was not statistically significant after multivariable adjustment. Although EDI provides a useful estimate of dietary Pb exposure, it does not directly indicate health risk. Future studies should integrate biomonitoring, species-specific bioavailability, and established toxicological risk metrics, such as the Target Hazard Quotient (THQ) and Margin of Exposure (MOE), to improve exposure assessment and better characterize the public health significance of dietary Pb exposure.

Several limitations should be considered when interpreting these findings. First, the cross-sectional design precludes causal inference, and the observed associations should not be interpreted as evidence of a causal relationship. Self-reported dietary behaviors and health symptoms may be subject to recall bias and misclassification, which could have introduced measurement error. Second, dietary Pb exposure was estimated using species-specific Pb concentrations and self-reported consumption patterns rather than biomarker-based measurements. Although this approach provides a practical estimate of dietary exposure, it cannot directly reflect internal Pb burden or individual toxicokinetics. Moreover, the estimated dietary Pb intake (EDI) does not account for inter-individual variability in gastrointestinal absorption, species-specific Pb bioavailability, food preparation practices, or other potential exposure sources, including drinking water, soil, household dust, occupational activities, household utensils, and tobacco use. Biomonitoring data, including blood lead levels (BLLs), were unavailable, and only a small proportion of participants (20.9%) had previously undergone BLL testing, indicating limited exposure surveillance within the study population. Third, environmental sampling was conducted during a single sampling period and may not capture seasonal variation in Pb concentrations. In addition, other potentially toxic metals, such as arsenic, cadmium, and mercury, were not measured. Therefore, co-exposure and residual confounding cannot be excluded, and the observed associations should be interpreted as reflecting potential combined environmental exposure rather than Pb-specific effects alone. Finally, because the prevalence of self-reported symptoms was relatively high (45.3%), the odds ratios may overestimate the strength of the associations and should be interpreted cautiously. Future prospective studies incorporating repeated environmental sampling, biomonitoring, multi-contaminant assessment, and quantitative exposure modeling are needed to improve exposure characterization and strengthen causal inference in lake-dependent communities.

## 5. Conclusions

This study identified female sex, lack of lead hazard training, consumption of more than 300 g of aquatic animals per meal, and long-term consumption exceeding 30 years as independent factors associated with self-reported health symptoms in this lake-dependent community. In contrast, consumption frequency and estimated dietary Pb intake (EDI) were not independently associated with symptom reporting after multivariable adjustment. These findings suggest that the amount and duration of aquatic animal consumption may be more important determinants of potential cumulative dietary Pb exposure than consumption frequency alone. However, these associations should not be interpreted as evidence that Pb was the cause of the reported symptoms. Rather, they suggest that aquatic animal consumption patterns may represent a potential pathway for dietary Pb exposure in this population. The reported symptoms are non-specific and may be associated with multiple environmental and behavioral factors, including possible dietary Pb exposure. Although Pb concentrations in aquatic animals remained below the current FAO/WHO Codex maximum level for fish muscle, compliance with this regulatory standard should not be interpreted as evidence of the absence of health risk. Rather, the findings suggest that long-term aquatic animal consumption may represent a potential pathway for cumulative dietary Pb exposure and warrant continued public health attention in communities that rely heavily on freshwater aquatic resources. These findings should be interpreted as identifying potential exposure pathways rather than demonstrating Pb-specific health effects, particularly given the possibility of co-exposure to other environmental contaminants. Accordingly, the observed symptom pattern should be interpreted as non-specific and potentially associated with multiple environmental and behavioral factors rather than Pb exposure alone. Future prospective studies incorporating biomonitoring, repeated environmental monitoring, and multi-contaminant exposure assessment are needed to clarify exposure–response relationships and strengthen causal inference. In the meantime, community-based health education, targeted dietary risk communication, and continued environmental and biomonitoring surveillance should be prioritized, particularly for populations with frequent or long-term consumption of freshwater aquatic foods, to reduce potential exposure and support informed dietary choices.

## Figures and Tables

**Table 1:** General characteristics of the study participants (*n* = 148).

Personal Characteristics	*n* (%)	
Gender		
Female	100	(67.6)
Male	48	(32.4)
Marital Status		
Single	42	(28.4)
Married	106	(71.6)
Age		
≤35 years	27	(18.2)
>35 years	121	(81.8)
Occupation		
Agriculture	51	(34.5)
Non-Agriculture	97	(65.5)
Educational background		
No formal education or primary education	63	(42.6)
Secondary education or higher	85	(57.4)
Smoking		
No	114	(77.0)
Yes	34	(23.0)
Alcohol consumption		
No	131	(88.5)
Yes	17	(11.5)
Annual health check-up		
No regular health check-up	64	(43.2)
Regular health check-up	84	(56.8)
History of Blood lead level (BLL) testing		
Never had BLL testing	117	(79.1)
Ever had BLL testing	31	(20.9)
Educational training on the hazards and toxic effects of lead		
No	33	(22.3)
Yes	115	(77.7)
Aquatic animals consumed		
>300 g/meal	68	(45.9)
≤300 g/meal	80	(54.1)
Frequency Aquatic animals consumed		
>1 meal/day	42	(28.4)
Rarely or never	106	(71.6)
Duration Aquatic animals consumed		
>30 years	70	(47.3)
≤30 years	78	(52.7)

**Table 2:** Types and prevalence of health impact symptoms among respondents (*n* = 148).

Health Symptoms	*n* (%)	Prevalence	95% CI
Musculoskeletal pain	118 (79.73)	0.797	(0.731, 0.862)
Joint and muscle pain	106 (71.62)	0.716	(0.642, 0.789)
Headache	87 (58.78)	0.587	(0.507, 0.668)
Irritability	81 (54.73)	0.547	(0.466, 0.628)
Fatigue	59 (39.86)	0.398	(0.319, 0.482)
Constipation	48 (32.43)	0.324	(0.248, 0.400)
Loss of appetite	34 (22.97)	0.229	(0.161, 0.298)
Nausea and vomiting	25 (16.89)	0.168	(0.107, 0.229)
Severe intermittent abdominal pain	22 (14.86)	0.148	(0.090, 0.206)
Pallor	13 (8.78)	0.087	(0.041, 0.133)
Lethargy	11 (7.43)	0.074	(0.031, 0.117)
Seizure	1 (0.68)	0.006	(0.0001, 0.037)

CI; confidence interval.

**Table 3:** Factors associated with any self-reported health symptoms among respondents (*n* = 148).

Factors	Symptom *n* (%)	No Symptom *n* (%)	Crude OR (95% CI)	Adjusted OR (95% CI)	*p*-Value
Gender ^a^					
Female	50 (50.00)	50 (50.00)	1.82 (0.897–3.708)	2.62 (1.099–6.249)	0.030 *
Male	17 (35.42)	31 (64.58)	1.00 (Ref)	1.00 (Ref)	
Age ^a^					
>35 years	54 (44.63)	67 (55.37)	0.86 (0.376–2.002)	0.76 (0.309–1.888)	0.560
≤35 years	13 (48.15)	14 (51.85)	1.00 (Ref)	1.00 (Ref)	
Educational level ^a^					
No formal education or primary education	28 (44.40)	35 (55.60)	0.94 (0.490–1.816)	0.71 (0.351–1.464)	0.381
Secondary education or higher	39 (45.90)	46 (54.10)	1.00 (Ref)	1.00 (Ref)	
Occupational ^a^					
Agriculture	27(52.90)	24 (47.10)	1.60 (0.810–3.172)	1.43 (0.682–3.008)	0.343
Non-agriculture	40(41.20)	57(58.80)	1.00 (Ref)	1.00 (Ref)	
Smoking ^a^					
Yes	18 (52.94)	16 (47.06)	1.49 (0.692–3.219)	1.97 (0.772–5.065)	0.155
No	49 (42.98)	65 (57.02)	1.00 (Ref)	1.00 (Ref)	
Alcohol consumption ^a^					
Yes	8 (47.06)	9 (52.94)	1.08 (0.394–2.986)	1.05 (0.321–3.472)	0.928
No	59 (45.04)	72 (54.96)	1.00 (Ref)	1.00 (Ref)	
Annual health check-up ^a^					
No regular health check-up	34 (40.48)	50 (59.52)	1.56 (0.812–3.016)	1.43 (0.697–2.943)	0.329
Regular health check-up	33 (51.56)	31 (48.44)	1.00 (Ref)	1.00 (Ref)	
History of Blood lead level (BLL) testing ^a^					
Never had BLL testing	58 (49.60)	59 (50.40)	2.40 (1.021–5.656) *	2.11 (0.748–5.952)	0.158
Ever had BLL testing	9 (29.00)	22 (71.00)	1.00 (Ref)	1.00 (Ref)	
Educational training on the hazards and toxic effects of lead ^a^					
Never	58 (50.40)	57 (49.60)	2.71 (1.161–6.341) *	3.39 (1.373–8.418)	0.008 *
Ever	9 (27.30)	24 (72.70)	1.00 (Ref)	1.00 (Ref)	
Aquatic animals consumed ^b^					
>300 g/meal	38 (55.90)	30 (44.10)	2.22 (1.150–4.314) *	2.47 (1.230–4.960)	0.011 *
≤300 g/meal	29 (36.30)	51 (63.70)	1.00 (Ref)	1.00 (Ref)	
Frequency Aquatic animals consumed ^a^					
>1 meal/day	20 (47.60)	22 (52.40)	1.14 (0.557–2.337)	1.15 (0.530–2.492)	0.724
Rarely or never	47 (44.30)	59 (55.70)	1.00 (Ref)	1.00 (Ref)	
Duration Aquatic animals consumed ^b^					
>30 years	38 (54.29)	32 (45.71)	2.00 (1.040–3.871) *	2.16 (1.065–4.398)	0.033 *
≤30 years	29 (37.18)	49 (62.82)	1.00 (Ref)	1.00 (Ref)	
Estimated dietary Pb intake among participants ^a^					
High EDI	35 (47.30)	39 (52.70)	1.18 (0.616–2.252)	1.15 (0.568–2.326)	0.698
Low EDI	32 (43.20)	42 (56.80)	1.00 (Ref)	1.00 (Ref)	

Hosmer–Lemeshow test [χ2 = 1.398, df = 5, *p* = 0.925]. OR = odds ratio; CI = confidence interval, Adjusted OR derived from multivariable logistic regression, *p* < 0.05 indicates statistical significance, Ref = reference group; BLL = blood lead level. ^a^ aOR adjusted for gender, aquatic animals consumed, smoking, training. ^b^ adjusted for age, training and smoking. * Statistically significant (*p* < 0.05).

## Data Availability

The data are not publicly available due to ethical and privacy restrictions but may be available from the corresponding author upon reasonable request.

## Supplementary Materials

The following supporting information can be downloaded at https://www.mdpi.com/article/10.3390/ijerph23070899/s1, Table S1: Descriptive statistics of lead (Pb) concentrations (mg/kg wet weight) in aquatic animal samples collected from five sampling sites around Nong Han Lake, Sakon Nakhon Province, Thailand.
