# Supplementary material for: Estimated Dietary Lead Exposure Through Aquatic Animal Consumption and Health Symptoms in a Lake-Dependent Community: Evidence from Nong Han Lake, Northeast Thailand
Source: Int J Environ Res Public Health. 2026 Jul 12;23(7):899. doi: 10.3390/ijerph23070899 (PMC13411962; doi:10.3390/ijerph23070899)
Supplement: Supplementary file 1 [file ijerph-23-00899-s001.zip › ijerph-4381659-supplementary.pdf]

Supplementary Table S1. Descriptive statistics of lead (Pb) concentrations (mg/kg wet weight) in aquatic animal samples collected from five sampling sites around Nong Han Lake, Sakon Nakhon Province, Thailand.

Sampling Site	Species	n	Mean (mg/kg)	SD (mg/kg)	Min (mg/kg)	Max (mg/kg)	FAO/WHO Limit (mg/kg)	Exceeds Limit?
**Site-1**	*Macrobrachium lanchesteri (River shrimp)*	4	0.0811	0.0044	0.0755	0.0858	**0.300**	No
	Oreochromis niloticus (Nile tilapia)	4	0.0419	0.0137	0.0291	0.0585	**0.300**	No
	Filopaludina martensi (Freshwater snail)	4	0.0849	0.0028	0.0819	0.0883	**0.300**	No
**Site-2**	*Macrobrachium lanchesteri (River shrimp)*	4	0.0888	0.0039	0.0858	0.0944	**0.300**	No
	Oreochromis niloticus (Nile tilapia)	4	0.0400	0.0162	0.0260	0.0625	**0.300**	No
	Filopaludina martensi (Freshwater snail)	4	0.0918	0.0138	0.0812	0.1108	**0.300**	No
**Site-3**	*Macrobrachium lanchesteri (River shrimp)*	4	0.0934	0.0064	0.0858	0.1013	**0.300**	No
	Oreochromis niloticus (Nile tilapia)	4	0.0533	0.0104	0.0457	0.0685	**0.300**	No
	Filopaludina martensi (Freshwater snail)	4	0.0967	0.0138	0.0819	0.1147	**0.300**	No
**Site-4**	*Macrobrachium lanchesteri (River shrimp)*	4	0.0804	0.0037	0.0777	0.0858	**0.300**	No
	Oreochromis niloticus (Nile tilapia)	4	0.0479	0.0076	0.0398	0.0575	**0.300**	No

	Filopaludina martensi (*Freshwater snail*)	4	0.0525	0.0241	0.0229	0.0819	**0.300**	**No**
**Site-5**	*Macrobrachium lanchesteri* (*River shrimp*)	4	0.0811	0.0045	0.0768	0.0858	**0.300**	**No**
	Oreochromis niloticus (*Nile tilapia*)	4	0.0988	0.0392	0.0585	**0.1510**	**0.300**	**No**
	Filopaludina martensi (*Freshwater snail*)	4	0.0770	0.0035	0.0743	0.0819	**0.300**	**No**

SD = standard deviation; Min = minimum; Max = maximum.

FAO/WHO = Food and Agriculture Organization of the United Nations / World Health Organization.

FAO/WHO maximum permissible limit for Pb in fish and aquatic products = 0.300 mg/kg wet weight.

* The highest recorded concentration was 0.1510 mg/kg (Oreochromis niloticus, Site-5), which remained below the permissible limit.
